# A modified high-throughput analysis of PLFAs in soil

**DOI:** 10.1016/j.mex.2018.10.022

**Published:** 2018-10-31

**Authors:** Stephanie Ellis, Karl Ritz

**Affiliations:** Nottingham University, Sutton Bonington Campus, Loughborough, Leicestershire, LE12 5RD, UK

**Keywords:** High-throughput PLFA analysis, Phospholipid fatty acids, Microbial community, Microbial ecology, Fatty acids

## Abstract

Microbial community profiling via phospholipid fatty-acid (PLFA) analysis is an insightful technique which elucidates the phenotypic structure of microbial assemblages within soil. Previous iterations of PLFA analysis have used large quantities of chemicals and can take extended periods of time to perform. Another barrier to the implementation of this method is the cost and availability of specialised machinery. We report on a high-throughput method which reduces both the time to extract PLFAs from soil and reduces the quantity of chemicals required.

## Graphical abstract

**Figure:**
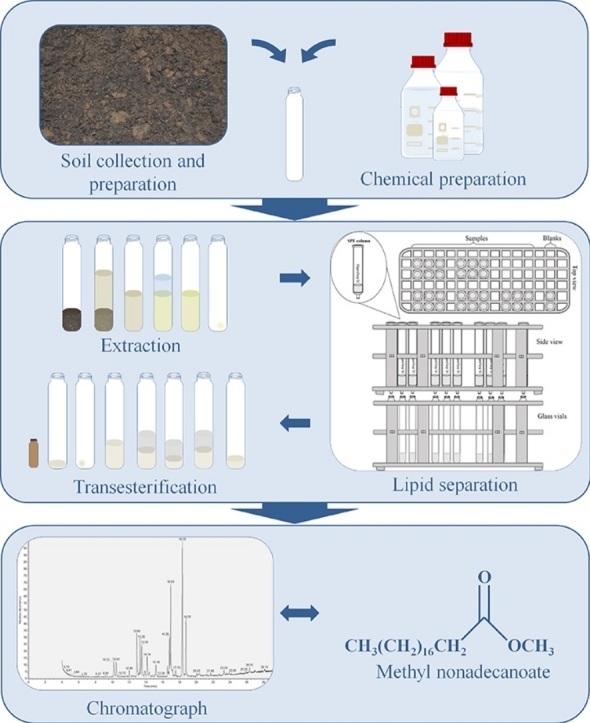

## Specifications table

**Table:** 

Subject area	Environmental Science
More specific subject area	Soil microbial ecology
Method name	High-throughput PLFA analysis
Name and reference of original method	J.S. Buyer, M. Sasser, High-throughput phospholipid fatty acid analysis of soils, Appl. Soil Ecol. 61 (2012) 127-130.
Resource availability	- Freeze-drier and pump (e.g. Alpha 2-4 LD, Christ, Germany; Vacuubrand 2.5, Germany). - Internal standard (e.g. 19:0 phosphatidylcholine, Avanti Polar Lipids, Alabaster, AL, US). - External standard (e.g. Bacterial Acid Methyl Ester, Supelco, Sigma Aldrich, US). - Solid phase extraction columns (e.g. HyperSep™ SI columns, 50 mg/1 ml, Thermo Scientific, US). - Centrifugal evaporator with vacuum pump, cold trap and rotor (e.g. miVac Duo Concentrator; miVac Duo Pump; miVac Speed Trap; rotor DRC-13100-032, GeneVac, UK). Nitrogen evaporation could be used instead of this equipment, if required. - GCMS, column and processing software (e.g. Trace GC Ultra and DSQ II, Thermo Scientific, US; ZB-FFAP, Phenomenex®,Macclesfield, UK; Xcalibur®, Thermo Fisher Scientific, US).

## Method details

### Overview

The analysis of phospholipid fatty acids (PLFAs) in soils can be labour intensive and costly. This can act as a barrier to implementation and use, potentially preventing the dissemination of high quality methods. A high-throughput PLFA method was recently developed [1], which can extract 95 samples (plus 1 blank) over a 2 day period while using significantly less chemicals. This is in contrast to other methods such as [2] and [3], which use a substantial volume of chemicals and take significantly longer to perform. Whilst this high-throughput method is an advancement on previous work, it uses products that are highly specific which may not be readily available. For example the use of a 50 mg silica gel SPE 96-well plate, a 1.5 ml multi-tier microplate, and a 96-well plate manifold. A centrifugal evaporator with cold trap and vacuum pump is also required, as well as rotors to fit the above microplates. The modifications in the method below mainly deals with substitutions for the 96-well plate and associated equipment. However, if a centrifugal evaporator is not available for use, then nitrogen evaporation could be utilised instead, as used in [2]. Furthermore, the point at which the internal standard is added has been altered to a later step, similar to that as observed in [2]. This allows for less internal standard to be used, reducing the quantity required overall and therefore reducing the cost of its use. Further advice regarding nitrogen evaporation and other considerations can be found in the section entitled “Notes on the protocol”.

### Reagents

Solvents should be HiPerSolv, HPLC or GC grade. All other reagents should be reagent grade. Deionised or Milli-Q^TM^ water should also be used, consistently, throughout. Reagents are stored with Teflon^TM^ lined lids, to reduce potential for plasticide contamination.

1 Bligh-Dyer extractant: 200 ml 50 mM K_2_HPO_4_ in *di*H_2_O, 500 ml methanol and 250 ml chloroform. Buffer to pH 7.4. ^TM^This should be made up fresh daily, or at least weekly.
2 Transesterification reagent: dissolve 0.561 g KOH in 75 ml methanol, add 25 ml toluene.
3 5:5:1 methanol:chloroform:H_2_O
4 0.075 M acetic acid
5 Internal standard: 19:0 phosphatidylcholine (Avanti Polar Lipids, US), dissolved in 1:1 chloroform:methanol (2 g l ^−1^). Stored neat at −20 °C and in a 4 °C fridge once made up (solution should last for 6 months).
6 External standard: Bacterial Acid Methyl Ester, supplied by Supelco (Sigma Aldrich, US), which contains 25 distinct PLFAs.
7 Chloroform
8 Methanol
9 Acetone
10 Toluene
11 Hexane

### Extraction

1 Freshly sampled soil should be placed in a −80 °C freezer immediately after acquisition, then subsequently freeze-dried before extraction of PLFAs. Ensure dry-weight is determined.
2 Weigh 2–3 g of freeze-dried soil into a 13 mm by 100 mm glass screw-cap test tube. Ensure that 1–2 blanks are also included.
3 Add 4.0 ml Bligh-Dyer extractant to the test tubes.
4 Seal the samples with a Teflon lined screw-cap lid.
5 Shake the samples by hand to ensure that the Bligh-Dyer extractant and soil mix properly.
6 Sonicate the test tubes in an ultrasonic bath for 10 min at room temperature. At this point ensure that the test tubes are not too closely packed such that sonication is not consistent between samples.
7 Shake the samples on an end-over-end shaker for 2 h (ensure that the caps are tightly sealed).
8 Centrifuge the samples for 10 min at 1500 rpm to separate the liquid and solid phases.
9 When removing the samples from the centrifuge minimise disturbance to the samples, as this could re-suspend some of the soil. In the event of re-suspension of the soil, centrifuge further before continuing.
10 Using a glass pipette with a rubber bulb attached, transfer the liquid phase to clean 13 mm by 100 mm glass screw-cap test tubes with a clean Teflon lined lid. Ensure that a new glass pipette is used for each sample. The rubber bulbs should be replaced fresh for each extraction run, or when clearly contaminated.
11 Add 1 ml of chloroform and 1 ml of *di*H_2_O (or Milli-Q H_2_O) to the new test tubes containing the liquid phase and seal the test tubes with Teflon lined screw caps lids.
12 Vortex the samples for 5 s to mix the phases.
13 Centrifuge the samples at 1500 rpm for 10 min to separate the two liquid phases.
14 Remove the upper phase of the sample using a glass pipette, with a rubber bulb and discard into an appropriate waste bottle. There will be an apparent meniscus between the two phases, which may contain dirt particles. Try to remove these particles as well during this step.
15 Evaporate the remaining lower phase of the sample at 30 °C in a centrifugal evaporator (e.g. miVac Duo Concentrator, Pump, Speed Trap and rotor DRC-13100-032, GeneVac, UK) for 40 min.
16 Store the samples at −20 °C overnight (or until ready for the next phase).

### Lipid separation

This section requires the conditioning of HyperSep^TM^ solid phase extraction (SPE) columns (Thermo Scientific, US) and subsequent lipid separation of the samples. The flow of reagents through the SPE columns can occur quickly. Additionally, the SPE columns, once wetted, must not be allowed to dry. Therefore some prior preparation of test tubes and samples is required.

**Fig. 1:**
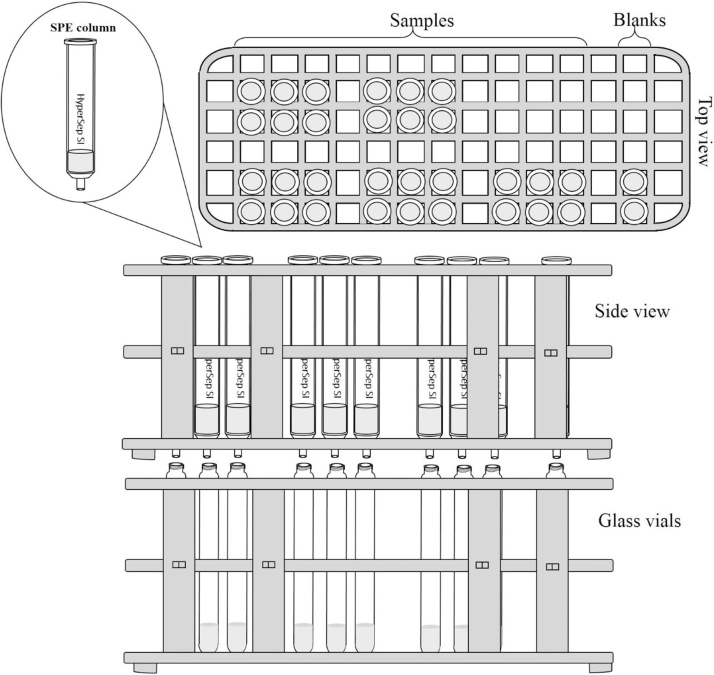
Example layout of solid phase extraction (SPE) columns and test tubes.
3 Place the SPE columns, containing 50 mg of silica per 1 ml column, into a test tube rack or other appropriate apparatus, which will allow them to be suspended over the ensuing waste beakers and test tubes. For ease, position the SPE columns to match the layout of the glass test tubes previously set out (Fig. 1).
4 Once the SPE columns are suitably positioned, place a large glass beaker beneath them. This beaker will be used to capture unwanted effluent. If a large glass beaker is not available, substitute with another container that is capable of containing the solvents used within this protocol.
5 Condition the well with three 1 ml aliquots of methanol (3 x 1 ml), followed by three 1 ml aliquots of chloroform (3 x 1 ml), capturing the effluent chemicals in the waste glass beaker.
6 Once the majority of the last chloroform addition has passed through the SPE column, transfer the re-suspended 1 ml of sample into the SPE columns using a glass pipette and rubber bulb. Ensure a new glass pipette is used for each sample. Pouring the sample to achieve this can be done instead and whilst it is more rapid, can be prone to spillage.
7 Allow the sample to pass through the silica within the column, capturing the effluent in the waste beaker.
8 Whilst the sample is passing through the column, rinse the glass test tube that previously contained the sample, with another 1 ml of chloroform. Transfer this to the SPE column and allow to pass through. Again, capturing the chemical effluent in a waste beaker.
9 Wash each well with 1 ml of chloroform, followed by 1 ml of acetone, again capturing the chemicals in the waste beaker.
10 Once all of the acetone has passed through the column and has been captured in the waste beaker, remove the waste beaker and replace with the previously prepared 13 mm by 100 mm glass test tubes.
11 To elute the phospholipids, add 0.5 ml of the 5:5:1 methanol:chloroform:H_2_O reagent to the SPE columns. Allow the solution to pass through and capture in the test tubes.
12 Add 0.1 ml of the internal standard to the test tubes.
13 Evaporate the solution at 70 °C for 30 min in a centrifugal evaporator, then evaporate at 37 °C, until dry. This should be done under vacuum.

1 In preparation, dissolve the dried samples in 1 ml chloroform and set to the side.
2 Similarly, set out 13 mm by 100 mm glass screw-cap test tubes in a test tube rack (without lids on), laid out to match the samples in use and set to the side. An example layout is shown in Fig. 1.

### Transesterification

1 Add 0.2 ml of transesterification reagent to each vial and seal the vials with Teflon lined lids.
2 Incubate the vials at 37 °C for 15 min.
3 Add a 0.4 ml aliquot of 0.075 M acetic acid and a 0.4 ml aliquot of chloroform to each test tube.
4 Vortex each test tube for 10 s.
5 Allow phases to settle and separate. This should not take more than a few minutes.
6 Using a pipette, or a glass pipette with rubber bulb attached, remove 0.3 ml of the lower phase and place into clean 13 mm by 100 mm glass test tubes.
7 Add another 0.4 ml of chloroform to the old glass test tube.
8 Again, vortex the glass test tubes for 10 s and allow the phases to separate.
9 Using a pipette, or a glass pipette with rubber bulb attached, again remove 0.4 ml of the lower phase and place into the new 13 mm by 100 mm glass test tubes (that contain the previously removed 0.3 ml of sample).
10 Evaporate the samples to dryness at room temperature in a centrifugal evaporator, held at vacuum.
11 Dissolve the samples in 75 μl hexane.
12 Transfer the samples to amber GC vials with 0.1 ml conical glass inserts and cap.
13 Store the GC vials at −20 °C until analysed using GCMS.

### GCMS analysis

The following is provided as an indicative set-up and procedure.

1 Inject 1 μl of sample into the GC, ensuring that the GC is set to split-less mode.
2 The column used in our work is a ZB-FFAP, supplied by Phenomenex^®^ (Macclesfield, UK). It is 30 m in length, with 0.25 mm inner diameter and 0.25 μm film thickness. Helium is used as a carrier gas, with a constant pressure of 18 psi. Initial oven temperature is 120 °C that is maintained for 1 min. The machine is programmed to increase in temperature (5 °C/minute) to 250 °C. This temperature (250 °C) is then maintained throughout the run.
3 The results are displayed as a chromatogram, showing the retention times of each compound. Xcalibur^®^ (Thermo Fisher Scientific, Waltham, US) is used to obtain and identify the PLFAs within each sample. The mass spectroscopy provides the ion profile of each compound, which aids in the identification of the peaks when compared to the internal and external standards.

### Validation

A sandy-loam soil was collected from the University of Nottingham Farm, Bunny Park (52.8607 °N, -1.1268 °W) and air-dried. It was then homogenised and passed through a 2 mm mesh sieve. Winter wheat (*Triticum aestivum* L.) *c.v.* Savannah and Line 85 was grown in the soil in polypropylene columns (170 mm by 60 mm) packed with 650 g of this soil, packed to a bulk density of approximately 1.1 g/cm^3^, and taken to field capacity (approximately 30% moisture content). Three replicates of one individual of each *c.v.* and a fallow control were established. All of these columns were then maintained in a growth chamber (12 °C during the 12 h night cycle and 18 °C during the 12 h day cycle) and watered weekly up to field capacity, for 1 month. In the last week, the columns were allowed to dry to below field capacity, to make sampling easier. The columns were then destructively sampled. Soil samples were frozen at −80 °C and subsequently freeze-dried for PLFA analysis. The protocol outlined above was followed. Mean soil moisture content across all treatments, at time of sampling, was 19.9 ± 3.4%.

Two soils, a sandy-loam and a loamy-sand, from the University of Nottingham Farm, Bunny Park (52.8607 °N, -1.1268 °W) were also tested. The soils were air-dried (to a moisture content < 5%), then homogenised and passed through a 2 mm mesh sieve. Four replicates were taken from each soil, then subsequently frozen at −80 °C and freeze-dried for PLFA analysis. The protocol outlined above was followed.

**Fig. 2:**
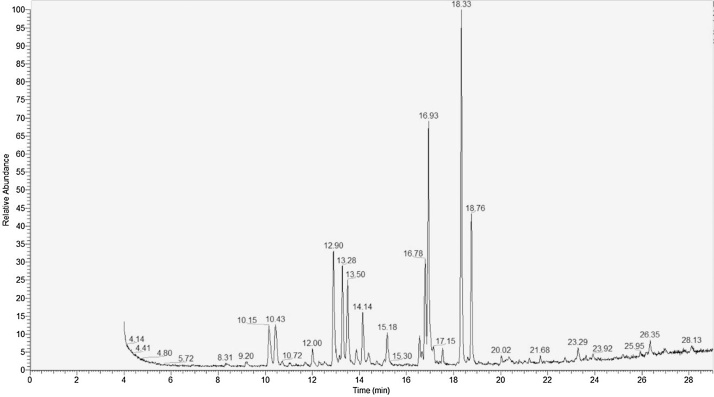
An example PLFA profile chromatograph produced from a sandy-loam soil using the adapted high-throughput PLFA protocol, the peak with a retention time of 18.33 min is the internal standard.

A range of 9 PLFAs were manifest and identified from the soils (Fig. 2; Table 1; Table 2). Assay-scale precision was acceptable, with the ranges in coefficient of variation being essentially the same between external standards and soil-based samples (Table 1).The sandy-loam and loamy-sand soils also exhibited the same PLFA profiles which are outlined in Table 2.

**Table 1:** Mean and associated coefficient of variation (CV) of % mol PLFA for external standard mix and soil communities in a sandy-loam soil, supporting different lines of winter wheat (n = 3).

	External Standard		Winter wheat; Savannah		Winter wheat; 85		Fallow	
Fatty acids	Mean (% mol)	CV	Mean (% mol)	CV	Mean (% mol)	CV	Mean (% mol)	CV
**i15:0**	11.2	5.95	18.0	2.53	18.4	1.60	18.2	3.77
**ai15:0**	10.1	4.66	15.0	1.60	14.9	1.82	14.3	3.52
**i16:0**	12.4	2.50	5.22	8.14	5.43	2.59	5.59	6.81
**16:0**	5.12	0.78	14.4	2.91	14.4	1.40	14.9	3.85
**16:1ω7*cis***	6.97	3.07	14.4	4.93	14.2	4.63	13.9	6.11
**3OH-12:0**	22.8	1.13	4.85	7.14	4.88	6.08	4.71	5.43
**18:0**	15.4	2.22	8.67	2.23	8.74	0.91	9.29	2.55
**18:2ω6*cis***	9.07	5.06	2.03	1.66	2.28	6.17	1.80	3.29
**19:0*cis***	6.97	6.10	17.4	3.90	16.9	3.32	17.3	1.69

**Table 2:** Mean and associated coefficient of variation (CV) of % mol PLFA for a loamy-sand and sandy-loam soil (n = 4).

	Loamy-sand soil		Sandy-loam soil	
Fatty acids	Mean (% mol)	CV	Mean (% mol)	CV
**i15:0**	10.6	3.84	8.84	7.62
**ai15:0**	6.87	3.46	7.07	7.79
**i16:0**	2.84	2.27	2.38	11.0
**16:0**	45.8	5.73	53.2	1.17
**16:1ω7*cis***	9.46	2.52	7.93	5.23
**3OH-12:0**	5.29	4.63	3.73	3.95
**18:0**	3.59	6.97	3.67	7.59
**18:2ω6*cis***	6.88	34.8	5.71	32.2
**19:0*cis***	8.67	2.86	7.52	6.97

### Notes on the protocol

1 All drying and evaporation steps should be performed in vacuo, using a centrifugal evaporator (with vacuum pump and cold trap). Where possible, all centrifugation steps should use the same centrifugal evaporator but with the vacuum pump turned off.
2 Evaporation under nitrogen can be used instead of a centrifugal evaporator [2]. If nitrogen evaporation is to be substituted, ensure that the flow of nitrogen over the meniscus of the sample is enough to cause disruption of the entire surface (but not to cause splash-back). This will ensure that all of the air is displaced from the surface of the sample, diminishing degradation of the sample. The stream of nitrogen (or placement) may also need to be altered during evaporation as the sample reduces in volume.
3 Specified 13 mm by 100 mm glass screw-cap test tubes are used throughout as they match the rotor (DRC-13100-032) for the miVac Centrifugal Concentrator. Approximately 30 samples and 2 blanks can be processed simultaneously with this rotor. When procuring such test tubes, some of them may not be of consistent size and will therefore not fit into the specified rotor. These can be used in the first step where Bligh-Dyer extractant is added, as an alternate centrifuge can be used with less strict constraints. There may be cheaper glass alternatives if using a different machine or using nitrogen evaporation. Additionally, for the last step, 13 mm by 100 mm *rimless* glass test tubes may be better to use as the diameter of the screw-cap variety can prevent the insertion of an electronic pipette. If using an electronic pipette to transfer the sample to the amber vial from a screw-cap test tube, the tip ejector arm of the pipette may need to be removed in order to fit.
4 Within this method, Teflon lined lids were used throughout. This was to prevent plasticide contamination. These have a finite usage and must be cleaned appropriately if re-used. Alternatively, normal lids can be bought and Teflon/PTFE tape can be used over the top of the glass tube. Whilst this is significantly cheaper, it is also very time consuming and cumbersome to deal with.
5 The quantity of internal standard to be added may vary depending upon the sample in question and the quantity of PLFA extracted.
6 Glass is used predominantly throughout as the chemicals involved can degrade plastic and lead to plasticide contamination of samples.
7 Glassware can be cleaned as outlined in [4].
